# HER2 Signaling and Breast Cancer Stem Cells: The Bridge behind HER2-Positive Breast Cancer Aggressiveness and Therapy Refractoriness

**DOI:** 10.3390/cancers13194778

**Published:** 2021-09-24

**Authors:** Serenella M. Pupa, Francesca Ligorio, Valeria Cancila, Alma Franceschini, Claudio Tripodo, Claudio Vernieri, Lorenzo Castagnoli

**Affiliations:** 1 Molecular Targeting Unit, Department of Research, Fondazione IRCCS Istituto Nazionale dei Tumori, AmadeoLab, Via Amadeo 42, 20133 Milan, Italy; alma.franceschini@istitutotumori.mi.it (A.F.); lorenzo.castagnoli@istitutotumori.mi.it (L.C.); 2 Medical Oncology Unit, Fondazione IRCCS Istituto Nazionale dei Tumori, Via Venezian 1, 20133 Milan, Italy; francesca.ligorio@istitutotumori.mi.it (F.L.); or claudio.vernieri@ifom.eu (C.V.); 3 Tumor Immunology Unit, University of Palermo, Corso Tukory 211, 90134 Palermo, Italy; valeria.cancila@unipa.it (V.C.); claudio.tripodo@unipa.it (C.T.); 4 IFOM the FIRC Institute of Molecular Oncology, Via Adamello 16, 20139 Milan, Italy

**Keywords:** breast cancer, cancer stem cells, full-length HER2, d16HER2 splice variant, p95HER2, stemness signaling pathways, drug resistance

## Abstract

**Simple Summary:**

Breast cancer (BC) is not a single disease, but a group of different tumors, and altered HER2 expression defines a particularly aggressive subtype. Although HER2 pharmacological inhibition has dramatically improved the prognosis of HER2-positive BC patients, there is still an urgent need for improved knowledge of HER2 biology and mechanisms underlying HER2-driven aggressiveness and drug susceptibility. Emerging data suggest that the clinical efficacy of molecularly targeted therapies is related to their ability to target breast cancer stem cells (BCSCs), a population that is not only self-sustaining and able to differentiate into distinct lineages, but also contributes to tumor growth, aggressiveness, metastasis and treatment resistance. The aim of this review is to provide an overview of how the full-length HER2 receptor, the d16HER2 splice variant and the truncated p95HER2 variants are involved in the regulation and maintenance of BCSCs.

**Abstract:**

HER2 overexpression/amplification occurs in 15–20% of breast cancers (BCs) and identifies a highly aggressive BC subtype. Recent clinical progress has increased the cure rates of limited-stage HER2-positive BC and significantly prolonged overall survival in patients with advanced disease; however, drug resistance and tumor recurrence remain major concerns. Therefore, there is an urgent need to increase knowledge regarding HER2 biology and implement available treatments. Cancer stem cells (CSCs) represent a subset of malignant cells capable of unlimited self-renewal and differentiation and are mainly considered to contribute to tumor onset, aggressiveness, metastasis, and treatment resistance. Seminal studies have highlighted the key role of altered HER2 signaling in the maintenance/enrichment of breast CSCs (BCSCs) and elucidated its bidirectional communication with stemness-related pathways, such as the Notch and Wingless/β-catenin cascades. d16HER2, a splice variant of full-length HER2 mRNA, has been identified as one of the most oncogenic HER2 isoform significantly implicated in tumorigenesis, epithelial-mesenchymal transition (EMT)/stemness and the response to targeted therapy. In addition, expression of a heterogeneous collection of HER2 truncated carboxy-terminal fragments (CTFs), collectively known as p95HER2, identifies a peculiar subgroup of HER2-positive BC with poor prognosis, with the p95HER2 variants being able to regulate CSC features. This review provides a comprehensive overview of the current evidence regarding HER2-/d16HER2-/p95HER2-positive BCSCs in the context of the signaling pathways governing their properties and describes the future prospects for targeting these components to achieve long-lasting tumor control.

## 1. Introduction

Breast cancer (BC) is the second most common cancer type, and a leading cause of death among women globally [1]. BC is a biologically complex and heterogeneous disease involving tumor cells and various other cell types, with different peculiar features, such as motility, plasticity, and stemness traits. Gene array technology has led to the identification of different BC molecular subtypes, and an improved understanding of their biological heterogeneity may lead to the development of more effective therapeutic strategies [2,3]. Accordingly, preclinical studies have demonstrated that efficient antitumor treatments must focus on targeting multiple distinct and nonredundant cell pathways in the different molecular BC subsets.

HER2 overexpression/amplification occurs in approximately 15–20% of BC cases and identifies a highly aggressive BC subtype [4]. Anti-HER2 treatment for HER2-positive (HER2+) BC has changed the natural history of this disease and has dramatically improved clinical outcomes in all disease stages, representing one of the greatest achievements in clinical oncology and providing clear evidence of the effectiveness of molecularly targeted therapeutics [4]. However, not all patients with limited-stage disease are cured [5], and HER2+ metastatic BC remains an almost invariably deadly disease. Thus, the identification and investigation of newer and more effective therapies represent an urgent medical need [6].

Over the past two decades, numerous studies have provided strong support that not all cells in tumors are equal and that tumors contain a dedicated small subpopulation of cells that possess the properties of normal stem cells. These cells, commonly referred to as cancer stem cells (CSCs) have been identified in a multitude of hematological and solid malignancies, including leukemia [7], colorectal [8], gastric [9], brain [10], and breast [11,12] cancers. Similar to the features of normal tissue-specific stem cells, the CSC theory postulates that the growth of tumors is fueled by limited numbers of CSCs dispersed in the bulk and at the edge of tumors. These peculiar cells have the ability to self-renew limitlessly and to differentiate into distinct cell lineages with the activation of various gene regulatory networks, which strongly suggests that they can initiate or maintain cancers [13]. Moreover, CSCs can switch between different phenotypic cell states and undergo epithelial-mesenchymal transition (EMT), a process in which cells lose their epithelial characteristics and gain mesenchymal traits; this ability allows CSCs to dedifferentiate and invade other tissues and organs. [14,15]. Of note, sufficient evidence has suggested that BC cells can spontaneously and stochastically interconvert between CSC and non-CSC states [16,17,18]. In accordance with the presence of this dynamic cell program, accumulating investigations have demonstrated that CSCs are significantly implicated in tumor progression, relapse, metastasis and resistance to therapies [18,19,20]. Hence, considering all these features, the CSC theory suggests that the design/development of more effective therapeutic strategies should be aimed at simultaneously targeting both the “root” of tumors and actively proliferating cancer cells, thus maximizing therapeutic efficacy against cancer [21]. In this context, emerging evidence has highlighted that the benefits of targeted therapies over traditional antiproliferative agents are associated with the capability of targeted therapies to effectively eradicate the CSC compartment [22,23]. Over the past two decades, several studies have shown that dysregulated HER2 expression/activation is considered a master regulator of EMT and CSC cell programs in both HER2+ (HER2-enriched/luminal B subtypes) and HER2-negative luminal BCs [24,25,26,27,28]. Additionally, at least part of the activity of anti-HER2 drugs, such as trastuzumab, a fully humanized anti-HER2 monoclonal antibody, and lapatinib, a dual tyrosine kinase inhibitor (TKI) that interrupts the HER2 and epidermal growth factor receptor (EGFR) pathways, lies in their selective targeting CSCs highly expressing HER2 either in breast [22,23,24,25,29] or gastric malignancies [30].

Several cancer-related signaling gene pathways are involved in the regulation of tumor cell activities among different molecular BC cell populations, including the HER2-enriched subset [31]. In particular, the dysregulation of Notch, Wingless (Wnt)/β-catenin, Sonic hedgehog, transforming growth factor-β/small mothers against decapentaplegic (TGF-β/SMAD), nuclear factor kappa-light-chain-enhancer of activated B cells (NFκB), and Janus Kinase/Signal Transducer and Activator of Transcription (JAK/STAT) signaling is directly implicated in the limitless self-renewal and cell differentiation abilities of both normal mammary stem cells (NMSCs) and CSCs [28,32,33]. Therefore, the elucidation of the pathways that specifically regulate the maintenance and survival of CSCs is important for the development of novel combinatorial therapies, and appears to be a high priority in the attempt to achieve long-lasting tumor control/eradication and eventually achieve a cure [34,35,36]. In this complex pathobiological scenario, we demonstrated that the d16HER2 splice variant, generated via the skipping of exon 16 in the extracellular domain of the full-length (FL)-HER2 receptor, constitutes a more oncogenic HER2 isoform heavily involved in HER2-driven tumorigenesis, aggressiveness, EMT/stemness cellular programs, and functional crosstalk with the Notch signaling pathway [36,37,38,39]. The co-existence of d16HER2 [36] with the other two naturally occurring HER2 splice variants (herstatin and p100) [40], with truncated HER2 fragments (p95HER2) [41,42] and with HER2 somatic mutations [43], greatly contributes to increasing the heterogeneity of HER2+ disease, thus limiting the effectiveness of therapy.

In this context, a subgroup of HER2+ BC patients with particularly poor clinical outcomes were found to express a variety of truncated HER2 fragments of 90 to 115 kDa [44,45] that have been found to drive metastasis [46] and refractoriness to trastuzumab [47]. In particular, 611-CTF is the most potent active truncated fragment of HER2 and heavily participates in in vivo BC progression and disease relapse by triggering multiple signaling cascades that lead to a continuous activation of the PI3K/Akt pathway [44]. Dysregulation of truncated p95HER2 induced by pharmacological in vitro and in vivo targeting of HER2+ models is linked to HER2-driven stemness (Table 1) [48,49,50].

In this study, we reviewed the current knowledge of the biological traits of HER2-/d16HER2-/p95HER2-driven breast CSCs (BCSCs) and their potential clinical relevance in HER2+ BC.

## 2. HER2+ BCSC Traits

### 2.1. Phenotypes of BCSCs

**Figure 1:**
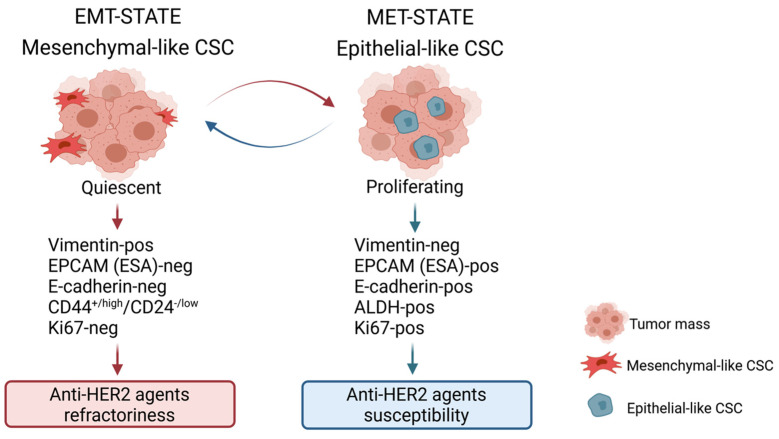
Characterization of BCSC states. Two different types of BCSCs have been identified: mesenchymal, quiescent cells with CD44^+/high^/CD24^−/low^ phenotype that are particularly refractory to anti-HER2 agent treatment and preferentially localized at the edge of the tumor mass (EMT-STATE); and epithelial, proliferating type cells characterized by ALDH activity (ALDH-pos) that are more susceptible to anti-HER2 agents and preferentially localized within the tumor mass. Image created by Biorender.com.

A common feature shared by CSCs of different oncotypes is the heterogeneous expression of distinct single markers or a combination of markers able to discriminate their specific phenotype from that of bulk tumor cells (Figure 1). To date, the most consistently used strategy for the identification of CSC phenotypes in the human mammary gland has relied on the surface expression of CD44 and CD24. CD44 is prevalently expressed on more progenitor-like cells, while CD24 is mostly expressed on more differentiated breast tissue cells; BCSCs are commonly identified as a CD44^high^/CD24^low^ population. Elevated expression of ALDH1, a polymorphic enzyme responsible for the oxidation of aldehydes and implicated in the detoxification of drugs and reactive oxygen species (ROS), is another biomarker of these cells, and can be assessed through the Aldefluor assay [51,52]. Of note, biomarker assessment is usually coupled with functional bioassays, including in vitro mammosphere formation efficiency (MFE) assays [53] and in vivo xenograft assays, the gold standard strategy for the assessment of bona fide CSCs [18].

#### Heterogeneity of CSC Phenotype/Properties

**Figure 2:**
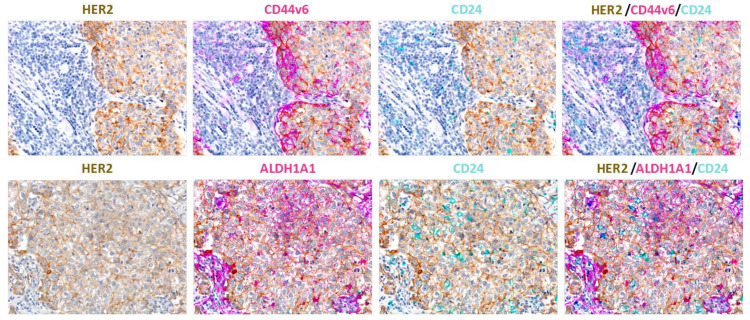
Triple-marker immunolocalization assessment of HER2 (brown signal), CD44v6 or ALDH1A1 (pink signal) and CD24 (cyan signal) in human breast cancer tissue samples. The results show different localizations of the two cell subsets. Original magnifications ×200.

Regarding the in vivo CSC bioassay, in a landmark study, in 2003, Al-Hajj and colleagues showed that human BC contains a defined subset of cells with the exclusive ability to form tumors in immunocompromised mice. Specifically, the authors provided clear evidence that 100 CD44^+/high^/CD24^−/low^ BC cells were sufficient to initiate primary mammary tumors, whereas bulk tumor cells with different phenotypes did not develop tumors [11]. More recent work by Liu and colleagues demonstrated that BCSCs are characterized by the plasticity to interconvert between two different phenotypic states. The first is a proliferative/cycling epithelial-like state acquired through mesenchymal-epithelial transition (MET) that is characterized by the expression of ALDH, E-cadherin and epithelial cell adhesion molecule (EPCAM); these cells are usually located more centrally within the tumor mass. In contrast, cells characterized by a more dormant/quiescent and invasive mesenchymal-like state, acquired through EMT, have a CD44^high^/CD24^low^ surface marker profile, express vimentin and N-cadherin and are localized at the tumor invasive front (Figure 1 and Figure 2) [15]. This biological scenario is similar across the different molecular subtypes of BC, even though the abundance of BCSCs and proportions of epithelial or mesenchymal BCSCs may differ among the distinct mammary tumor subgroups. Immunohistochemistry (IHC) analyses highlighted that the highest proportion of ALDH1-expressing epithelial CSCs is observed in the HER2+ BC subtype, and consistently, ALDH1 expression was found to be associated with a poor prognosis in a cohort of 203 primary BC patients [54]. On the other hand, CD44^high^/CD24^low^ cells were found to be more frequently enriched in the basal-like BC subtype than in any other BC molecular subset [55]. In the context of HER2-nonamplified luminal BC, Duru et al. revealed that a subpopulation of HER2+ CD44^high^/CD24^low^ BCSCs isolated from radioresistant BC MCF7 cells displayed enhanced ALDH activity, aggressiveness, tumor sphere formation, and in vivo tumorigenesis compared with their counterpart HER2-negative CD44^high^/CD24^low^ BCSCs [56], still supporting the notion that HER2 is a key BCSC biomarker that identifies a cell subpopulation particularly resistant to conventional anticancer therapies. Notably, the same authors found that ALDH1 upregulation in the HER2+ BCSC compartment of luminal BC was attenuated by HER2 inhibition via specific small interfering RNA (siRNA) or trastuzumab treatment [56]. Additionally, in the same study, the analysis of 40 BC specimens revealed that the HER2+ CD44^high^/CD24^low^ BCSC population was more frequently detected in recurrent BC than in primary BC [56], suggesting that the CD44^high^/CD24^low^ compartment is particularly refractory to anticancer therapy. In this context, more importantly, it is possible to argue that HER2-targeting agents such as trastuzumab or lapatinib may target CSCs in patients with HER2 nonamplified BC. Globally, based on the current knowledge, several published investigations support the idea that the CD44^high^/CD24^low^ and ALDH1+ stem cell-like phenotypes can be used to identify BCSCs with distinct levels of differentiation; the former profile is more related to basal-like BC (basal/HER2), while the latter reflects HER2-overexpressing tumors originating from luminal progenitors (luminal/HER2) [22].

### 2.2. The d16HER2 Splice Variant: The Key HER2 Biomarker Regulating HER2-Driven Stemness

**Figure 3:**
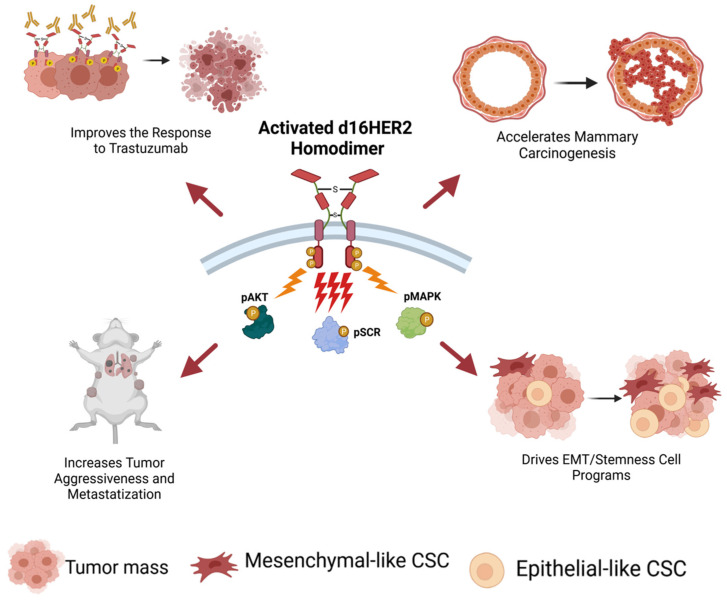
Schematic of the pathobiological features driven by constitutively activated d16HER2 homodimers (pd16HER2D). Multiple downstream signaling pathways, including the mitogen-activated protein kinase (MAPK), protein kinase B (AKT), and proto-oncogenic tyrosine-protein kinase Src (SRC) pathways, are activated. Image created by Biorender.com.

In 1998, an alternative splicing form of the human HER2 gene lacking exon 16, referred to herein as d16HER2, was detected by Kwong and Hung in human BCs [57]. d16HER2, expressed on the tumor cell plasma membrane, forms constitutively active homodimers (pd16HER2D) able to activate multiple downstream oncogenic signal transduction pathways and represents a constitutively active form of FL-HER2 [36]. In particular, as shown in several studies using ad hoc engineered tumor cell models, this alteration induces a greater transformation activity than FL-HER2 [57,58,59,60,61,62,63]. Of note, the first clinical evidence that linked the expression of the d16HER2 variant with tumor progression and metastasis was reported in 2009 by Mitra and colleagues [59]. Specifically, they showed that approximately 90% of HER2+ BC that coexpressed the d16HER2 variant developed locally disseminated node-positive BC versus those that were d16HER2-negative [59]. Comparative pathobiological analyses between transgenic (tg) mouse models expressing the human d16HER2 splice variant and the FL-HER2 receptor revealed the existence of a crucial biological link between the expression/activation of d16HER2 homodimers and HER2-driven mammary tumorigenesis and progression in the form of their direct functional signaling crosstalk with activated SRC kinase (pSRC), highlighting the existence of the pd16HER2D/pSRC signaling axis [37]. Consistent with the mouse results, we found that HER2+ BC patients with a highly activated pd16HER2D/pSRC signaling axis, which was molecularly identified by the presence of a genomic profile characterized by the significant enrichment of gene pathways related to hypoxia, tumor metastasis and cell motility (termed activated d16HER2 metagene), derived the greatest benefit from trastuzumab treatment [37]. We further examined the impact of d16HER2 expression on the stemness of HER2+ BC cells in patients, and found that BC patients with high expression levels of the activated-d16HER2 metagene exhibited significant enrichment of the Notch family and signal transducer genes, compared with those with low expression of the metagene [37]. Taken together, these preliminary findings indicate the significant enrichment of d16HER2+ BCSCs in HER2+ BC patients with high versus low d16HER2 metagene expression [37]. Consistently, subsequent functional analyses of mammary tumor cell lines derived from spontaneous mammary lesions arising in tg d16HER2 and FL-HER2 mice demonstrated that d16HER2+ cells exhibited significantly higher MFE and in vitro secondary and tertiary mammosphere formation and displayed greater in vivo tumor engraftment in serial dilution conditions than their FL-HER2+ cell counterparts, highlighting their CSC-like traits [36,38] (Figure 3). Of note, ad hoc molecular analyses provided evidence of the significant enrichment of Wnt, Notch and EMT gene expression in d16HER2+ cells compared with FL-HER2+ cells [36,38]. Furthermore, d16HER2+ mammary tumors exhibited a higher frequency of CD29^high^/CD24^+^/SCA1^low^ cells, a peculiar population enriched in murine mammary stem cells [64], than their FL-HER2+ cell counterparts [38]. The above mouse findings were validated in human cells via the engineering of HER2-negative MCF-7 and T47D BC cells with the human d16HER2 and FL-HER2 transgenes. Again, comparative analyses highlighted the significant enhancement, not only of the MFE, but also of the fraction of ALDH+ cells in the d16HER2-infected cells versus the FL-HER2-infected cells, thus unveiling a direct action of the d16HER2 splice variant in human HER2+ BCSCs [38]. Overall, these observations prompted us to speculate that the d16HER2 variant induces a key oncogenic signal that has a significantly stronger impact on HER2-driven BC EMT and stemness than its FL-HER2 counterpart.

### 2.3. Truncated p95HER2 Fragments: HER2 Biomarkers Regulating Trastuzumab Resistance

A subgroup of HER2+ BC cases, representing approximately 30% of HER2+ BC cases, co-expresses a heterogeneous collection of truncated HER2 CTFs, collectively called p95HER2, that lack the HER2 extracellular domain (ECD) and display distinct oncogenic potency [41,65]. p95HER2 fragments arise through at least two different mechanisms: proteolytic shedding of the FL-HER2 ECD [66,67] and translation of the HER2 mRNA from internal initiation codons [68]. Shedding of the ectodomain of HER2 generates a membrane-anchored p95HER2 fragment identified as 648-CTF [69], whose oncogenic activity was reported to be comparable to that of FL-HER2 [65]; on the other hand, the alternative initiation of translation generates two p95HER2 fragments known as the membrane-anchored oncogenically active fragment 611-CTF and the intracellular, soluble, inactive 687-CTF. Specifically, the expression of the 611-CTF fragment leads to rapid and potent activation of different signaling cascades due to the constitutive generation of homodimers maintained by intermolecular covalent bonds [44], i.e., the same mechanism of activation that we demonstrated for the d16HER2 splice variant [37]. Truncated 611-CTF, whose oncogenic effect is higher than that of FL-HER2, was found to selectively control the expression of genes not regulated by FL-HER2 that are causally involved in metastatic BC progression [44]. In this context, two clinical studies reported that 611-CTF positivity in primary BC patients is associated with poor prognosis [45] and nodal metastasis [46]. Furthermore, truncated p95HER2 has been shown to have a peculiar structure, and both preclinical [47] and clinical investigations in the metastatic HER2+ BC setting have provided evidence that CTFs are relevant biomarkers of trastuzumab therapy resistance [47,70]. However, these tumors respond to lapatinib, as do p95HER2-negative tumors [71]. In 2016, Kim and colleagues reported that treatment with disulfiram (DSF) (Table 1), an anti-alcoholism drug with antitumor activity [72], in the presence of copper (Cu)-induced apoptosis, was associated with a marked decrease in HER2 and truncated p95HER2, phospho-HER2 (p-HER2), HER3, phospho-HER3 (p-HER3) and phospho-Akt (p-Akt), which was simultaneously accompanied by decreased ALDH1 activity and decreased MFE. Of note, the inhibition of MFE by DSF/Cu treatment was coupled with a concomitant downregulation of transcripts of the key stemness-related transcriptional factors Nanog, Oct4 and Sox2 in HER2-positive SKBR3 and BT474 cells [48]. Moreover, to verify whether HER2 was involved in the regulation of BCSC properties, the cells were treated with trastuzumab, which was found to achieve a marked decrease of ALDH1A levels and MFE. Additionally, xenograft tumors derived from trastuzumab-sensitive BT474 cells pretreated in vitro with DSF/Cu exhibited a significant decrease of tumor growth associated with the downregulation of p95HER2 and ALDH1A1 protein content compared to untreated tumors [48]. In 2018, the same authors reported that treatment with flubendazole (Flu) (Table 1), a potent anthelmintic agent with antiproliferative activity in various types of cancer [73], induced apoptosis concomitant with a significant decrease in the activation status of p95HER2, HER2, HER3, Akt and HER2/HER3 heterodimers in both trastuzumab-susceptible (BT474 and SKBR3) and trastuzumab-refractory (JIMT-1 and MDA-MB-453) HER2+ BC cells [49]. Furthermore, flu therapy effectively targeted BCSC-like properties by decreasing ALDH1 activity and the CD44^high^/CD24^low^ cell population and suppressing MFE. Of note, Flu administration caused relevant tumor inhibition in JIMT-1 xenografts by inducing apoptosis coupled with the downregulation of BCSC-related biomarkers, as well as p95HER2 expression [49]. HER2 is one of many oncoproteins that is a client of heat shock protein 90 (HSP90), a chaperone protein that assists other proteins in folding properly and plays crucial roles in cellular homeostasis [74]. Consistent with these important biological features, HSP90 inhibition represents an attractive strategy for the treatment of neoplasms and other diseases [74]. In this context, the C-terminal HSP90 inhibitor NCT-547 (Table 1) was found to: (1) induce apoptosis and target HER2 signaling; (2) promote the degradation of FL-HER2 and truncated p95HER2 in cells properly engineered to ectopically express both molecules; (3) inhibit tumor growth in trastuzumab-resistant JIMT-1 xenografts; and (4) reduce ALDH1 activity, the CD44^high^/CD24^low^ subset and MFE, both in vitro and in vivo [50]. Even if no causal direct functional link between p95HER2 expression/activity and regulation of HER2+ BCSC features was clearly provided by the authors of these studies [48,49,50,74], we argue that truncated p95HER2 fragments could directly influence HER2 BC stemness.

## 3. Therapeutic Approaches for Targeting HER2+ BCSCs

### 3.1. Targeting HER2 Activity

As mentioned in the Introduction section, different pivotal studies have documented the prominent efficacy of trastuzumab and lapatinib in their selective targeting of BCSC population highly expressing HER2 [23,24,25,29,75,76,77].

#### 3.1.1. Trastuzumab-Driven Therapeutic Effects

In 2008, Korkaya et al. demonstrated, for the first time, that HER2 overexpression drives mammary tumorigenesis as well as tumor growth and invasion, through its effects on normal and malignant mammary stem cells. In particular, the authors showed that HER2 upregulation was directly correlated with an enrichment of ALDH+ spheres, and with the enhanced expression of stemness-related molecules, such as octamer-binding transcription factor (Oct)3/4, Notch1 and 2, Jagged1 and glioma-associated oncogene (Gli1), through activation of the downstream PI3K/Akt signaling pathway [24]. Additionally, the authors observed that trastuzumab decreased the ALDH+ subset by more than 50% in HER2+ BC cell lines through the inhibition of PI3K/Akt signaling [24]. Subsequently, Magnifico and colleagues notably defined that the remarkable clinical efficacy of the anti-HER2 agent trastuzumab was related to its ability to target CSCs and provided further solid preclinical evidence for an important role played by HER2 itself in the regulation and maintenance of the CSC population [25]. The researchers also showed that the effects were dependent on the higher HER2 expression levels in HER2+ BCSCs than in the cells forming the bulk of the tumor. The authors also proposed that Notch genes regulate HER2 expression, thereby acting as a signaling pathway sustaining HER2+ BCSC survival, self-renewal, tumorigenesis, and invasion [25]. In this context, Koschorke et al. revealed that β-phenethyl-isothiocyanate (PEITC), one of the most studied isothiocyanates, may act as a potent antitumor agent that targets HER2+ breast (BT474, SKBR3 and HCC1954) and ovarian (SKOV3) CSCs by decreasing their ALDH+ cell compartments, and they found that PEITC is able to reduce Notch 1 activation by acting as a negative modulator of crosstalk between HER2 and the Notch pathway [78]. Additionally, PEITC, alone or in combination with trastuzumab, significantly inhibited the MFE of d16HER2+ cells and reduced spontaneous tumor development in d16HER2 tg mice [78].

More recently, the Martin-Castillo study revealed the new concept that the differential enrichment of trastuzumab-responsive ALDH+ CSCs versus trastuzumab-refractory CD44^high^/CD24^low^ cells could explain both the clinical behavior and the efficacy of primary trastuzumab in each distinct intrinsic molecular subtype of HER2+ BC [29].

#### 3.1.2. Lapatinib-Driven Therapeutic Effects

Based on the notion that chemotherapy treatment increases the percentage of CD44^high^/CD24^low^ cells [75], Li and colleagues demonstrated that while the treatment of a “mixed” BC patient cohort with chemotherapy increased the percentage of CD44^high^/CD24^low^ cells and induced the formation of mammospheres, the combinatorial administration of chemotherapy plus lapatinib in specific HER2+ BC cases did not lead to the accumulation of CD44^high^/CD24^low^ BCSCs, indicating that the mesenchymal BCSC compartment is not totally refractory to lapatinib activity [75]. The findings of this study were encouraging and specifically suggested that inhibition of key regulatory pathways responsible for self-renewal, i.e., the HER2 and EGFR pathways, can augment the effects of conventional therapy and improve clinical outcome [75]. Subsequently, in support of the above data, Gong and colleagues demonstrated that the treatment of HER2+ epirubicin-resistant SKBR3 cells and adriamycin-resistant luminal A MCF7 cells with lapatinib or salinomycin, an inhibitor of oxidative phosphorylation (OXPHOS) that efficiently targets both CSCs and therapy-resistant cancer cells, significantly reduced the MFE by approximately 10–100-fold in the CD44^high^/CD24^low^ BCSC subset and, in parallel, decreased the percentage of ALDH+ cells, enhancing the sensitivity of BC cells to chemotherapy by approximately 30-fold [76]. In light of the increased numbers of CSCs in high-grade ductal carcinoma in situ (DCIS), a preinvasive lesion characterized by HER2 amplification/overexpression in up to 60% of cases [79]. Farnie et al. showed that lapatinib effectively targeted HER2+ DCIS-CSCs in distinct in vitro models by suppressing their proliferation [77]. Of note, the translational relevance of these findings strongly implies that the use of lapatinib in high-risk DCIS patients has the potential to reduce the risk of recurrence and the progression of DCIS to invasive disease [77].

Moreover, in accordance with landmark clinical studies suggesting that HER2 inhibition may be beneficial in the adjuvant setting for HER2 nonamplified BC patients [80,81], Lee and colleagues documented that the pharmacologic inhibition of EGFR, HER2, or both receptors reduced BCSC survival and self-renewal both in vitro and in vivo and increased BCSC sensitivity to ionizing radiation also in HER2-low BC [82].

Recently, it was reported that carbon-ion beams, which are characterized by a well-localized energy deposition, have enhanced activity in killing radioresistant cancer cells compared to conventional X-ray radiotherapy [83]; when combined with DNA damaging drugs, these beams are able to promote the in vitro eradication of triple-negative BCSCs [84]. Moreover, relatively low doses of carbon-ion beam irradiation combined with lapatinib have promising advantages over carbon-ion beam or conventional X-ray irradiation alone in targeting putative HER2+ BCSCs derived from both BT474 and SKBR3 cell lines, because they induce consistent DNA damage, increased apoptosis and autophagy, and subsequent cell death [85].

### 3.2. Targeting the Interplay between Stemness-Related Signaling Pathways and HER2+ BCSCs

Key studies have highlighted that the targeting of molecular drivers controlling CSC maintenance and plasticity, in combination with the administration of gold-standard treatments, may improve therapeutic control and patient outcomes, paving the way for a long-lasting therapeutic response; various treatments attempting to decrease/eradicate HER2+ BCSCs to overcome drug resistance over the course of treatment are emerging (see Table 1). In this context, given the heterogeneity of human BC, preclinical studies have demonstrated that efficient antitumor strategies must focus on the simultaneous targeting of multiple nonredundant, stem cell-specific pathways that regulate the stemness of malignant cells [28,35,86]. In particular, developmentally conserved signaling pathways such as Notch, Wnt, and Sonic Hedgehog, together with various other signaling pathways (see 3.2.4. paragraph), participate in crosstalk with HER2 and are considered critical nodes regulating the formation, survival, and proliferation of CSCs, as well as drug resistance in HER2+ BC. Of note, each of these pathways can crosstalk with each other, contributing to the establishment of a very complex cancer network scenario, whose targeting represents a crucial clinical challenge.

#### 3.2.1. Notch/HER2+ BCSC Crosstalk

Among the various stemness-related signaling pathways, canonical Notch signaling has been widely indicated to have bidirectional crosstalk with the HER2 circuitry, mainly due to the pivotal demonstration that the HER2 promoter contains Notch-binding sequences [87]. Several studies have described how the active interplay between Notch and HER2 controls the fate of BCSCs, impacting tumor development, drug resistance, recurrence and metastatic progression [88,89]. Consistently, Osipo and coauthors revealed that the enhanced activation of Notch signaling in distinct HER2+ cell models in response to trastuzumab treatment might be responsible for therapy resistance, which could be prevented or reversed by dual inhibition using trastuzumab and Notch1 siRNA or a γ-secretase inhibitor (GSI) [90]. Subsequently, it was reported that combined inhibition of the Notch and HER2 signaling pathways with GSI plus trastuzumab in orthotopic HER2+ BC xenografts could prevent or significantly reduce tumor growth by reversing trastuzumab resistance in resistant HER2+ cell models [91]. Several studies have documented that BC mortality is principally due to tumor recurrence that arises from a reservoir of residual tumor cells that pass through a dormant phase prior to relapse [92]. Specifically, Abravanel and colleagues performed a computational interrogation of data from 4463 BC patients across 17 individual datasets and genetically engineered mouse models for HER2/neu-targeted therapy; the researchers clearly elucidated that Notch signaling remains activated in a subset of dormant tumor cells after HER2 inhibition and promotes BC recurrence [93]. Taken together, these data suggest that Notch may provide a critical compensatory signaling pathway through which HER2+ dormant tumor cells survive after therapy, and that this pathway can be effectively targeted pharmacologically in the neoadjuvant setting with Notch inhibitors in combination with HER2/neu-targeted therapies to prevent BC recurrence [93]. Furthermore, based on the notion that the upregulation of Notch1 and the canonical Notch ligand Jagged1, one of five known proteins that interact with the four Notch receptors in mammals, predicts poor outcome, Shah and coauthors recently demonstrated that inhibition of HER2 with lapatinib increased the levels of Jagged1 on the cell surface of HCC1954 and MCF7-HER2 cells, increased the expression of the downstream Notch target genes HEY2 and HES4, increased the percentage of cells in the S-phase of the cell cycle, and increased the survival of BCSCs [94]. In this scenario, they found that higher Jagged1 membrane staining predicted poor overall survival of women with primary invasive HER2+ BC suggesting that the sequential blockade of HER2 and Jagged1 or Notch may be more effective than simultaneous blockade in terms of preventing drug resistance and tumor progression [94].

#### 3.2.2. Wnt/HER2+ BCSC Crosstalk

The interplay occurring between Wnt/β-catenin signaling activation and HER2 was first reported by Wu et al. [95]. The researchers found that WNT3 overexpression in trastuzumab-resistant BC cells increased the nuclear expression of β-catenin and promoted an EMT-like process with increased N-cadherin, Twist, and Slug expression and simultaneously decreased E-cadherin expression [95]. On the other hand, knockdown of WNT3 by siRNA restored the cytoplasmic expression of β-catenin and decreased the expression of EMT markers, suggesting that an EMT-like process may be one of the crucial CSC-related traits of cells that acquire trastuzumab resistance [95]. Likewise, high expression of cyclin-dependent kinase 12 (CDK12), a cyclin-dependent kinase involved in DNA damage repair and coamplified with HER2 at chromosome 17, was found to be associated with disease recurrence and poor survival; this high CDK12 expression activates WNT1- and WNT2-mediated Wnt/β-catenin signaling cascades to enhance the stemness of HER2+ BC and, in turn, induces trastuzumab resistance [96]. Specific CDK12 kinase inhibition with dinaciclib (Table 1) represents a broadly effective therapy against different types of HER2+ BC models [96]. Pharmacological inhibition of Wnt signaling using PKF118-310 was found to eradicate BCSCs, leading to durable HER2+ mammary cancer remission in proper HER2+ preclinical BC models [97]. Many inhibitors specifically targeting the Wnt signaling pathway are currently under investigation in combination with anti-HER2 agents for the treatment of BC patients [35].

#### 3.2.3. Sonic Hedgehog/HER2+ BCSC Crosstalk

On the other hand, there is very little published information describing crosstalk between HER2 and the Sonic Hedgehog (Shh) signal transduction pathway. In one study, tissue microarray data from 334 “mixed” human BCs revealed that Shh expression was higher in the HER2+ subgroup than in other BC subtypes, while Gli1 expression was enhanced in the HER2+ luminal B subtype [28].

#### 3.2.4. Crosstalk of Other Signaling Pathways with HER2+ BCSCs

As mentioned above, in addition to stemness-related developmentally conserved signaling pathways, various other signaling pathways are involved in crosstalk with HER2+ BCSCs. In particular, accumulating evidence also indicates that functional crosstalk between HER2 and TGF-β/SMAD signaling results in increased cancer cell proliferation, survival and invasion, accelerated metastasis in animal models, resistance to chemotherapy and HER2-targeted therapy, and the upregulation of BCSCs [22,23]. In line with this, the findings reported in 2011 by Chow et al. suggested that the simultaneous blockade of the TGFβ/HER2 axis may significantly enhance the efficiency of conventional therapies in HER2+ BC patients [98]. In a recent study, Chihara et al. revealed that continuous stimulation with TGF-β induces the robust phosphorylation of SMAD3, which subsequently induces an increase in the CD44^+^/CD24^−/low^ population in different HER2+ BC cell lines, causing resistance to anti-HER2 drugs [99]. Specifically, SIS3 (Table 1), a specific SMAD3 inhibitor, might reduce resistance by regulating the TGF-β signaling pathway and restore sensitivity to trastuzumab [99]. A83-01 (Table 1), another small molecule inhibitor, was suggested to modulate the TGF-β/SMAD pathway and prevent the development of a mesenchymal phenotype in HER2+ BC cells, thus enhancing the cell response to trastuzumab treatment [100].

## 4. Molecular Mechanisms Involved in the Refractoriness of BCSCs

Pharmacological blockade of oncogenes is effective but transient, and current antitumor therapies fail to prevent cancer relapse and metastasis. Indeed, although the availability of a plethora of promising anti-HER2 compounds has improved the prognosis of the HER2+ BC subtype, tumor recurrence due to de novo or acquired refractoriness of tumor cells to current treatments remains a major concern. In recent years, accumulating data have provided evidence of direct (CSC-intrinsic) and indirect (CSC-extrinsic) effects, linking anti-HER2 therapy resistance to BCSCs [23]. In particular, two distinct HER2+ BCSC compartments, i.e., the basal/HER2 and luminal/HER2 compartments (see Section 2.1), are characterized by different modes of trastuzumab refractoriness; indeed, mesenchymal CD44^high^/CD24^low^ BCSCs have been considered to be responsible for de novo/intrinsic trastuzumab resistance, while epithelial-like ALDH1A+ BCSCs, which are enriched in the luminal/HER2 subtype, are considered responsible for acquired trastuzumab resistance [22]. Of note, the crucial mechanism of de novo resistance to trastuzumab is linked to HER2-ECD shedding by zinc-containing metalloproteinases, including A disintegrin and metalloproteinases (ADAM) and matrix metalloproteinases (MMPs) family members [44,66,101]. In this context, ADAMs 10 and 17 have been reported as the major proteolytic enzymes involved in HER2 shedding [102], and, of note, higher ADAM10 levels were reported to be significantly correlated with poorer relapse-free survival in a cohort of HER2 positive breast cancer patients [103], suggesting the candidacy of ADAM10 as a potential target to bypass trastuzumab resistance (Table 1).

Recent studies indicate that numerous molecular mechanisms protect BCSCs from chemotherapy, radiotherapy and targeted therapy-induced death stimuli, thus favoring their enrichment [33,86]. Globally, the ability of BCSCs to survive under stressful conditions is correlated with the protection of genome integrity fueled by prompt activation of DNA damage sensor and enhancement of repair machinery [104]. Several other investigations in different BC cohorts have demonstrated that the reduced antitumor therapy efficacy in BCSCs is also related to enhanced expression of antiapoptotic proteins, such as members of the Bcl-2 family [86], immune evasion [33] and the overexpression of ATP-binding cassette (ABC) transporters such as ABCG2 (BCRP) and P-glycoprotein, which facilitate drug efflux out the tumor cells [86]. Compelling evidence also suggests that stem-like features can be acquired as a result of metabolic shifts. Indeed, metabolic insults have been revealed to induce reprogramming that redirects normal and tumor cells toward less-differentiated CSC cellular states, a phenomenon termed “metabostemness” that has been recently proposed as a new cancer hallmark [105]. In fact, some altered/dysregulated metabolic enzymes involved in glycolysis, OXPHOS, the tricarboxylic acid cycle and mitochondrial fatty acid oxidation can act as oncogenic drivers promoting tumorigenesis and tumor aggressiveness, and targeting these factors may represent a successful strategy [105]. Accordingly, salinomycin, an OXPHOS inhibitor, was demonstrated to kill BCSCs [106].

In addition to the above-described intrinsic mechanisms of therapy resistance acting in CSCs, EMT is considered a crucial process in mediating refractoriness to conventional treatments. The transition from an epithelial to a mesenchymal phenotype requires the dynamic and coordinated dysregulation of the expression/activity of several genes and transcriptional regulatory factors including Snail, Slug, Twist, zinc finger E-box-binding homeobox-1 (ZEB1), and ZEB2; several stemness-related signaling circuits such as Notch, Wnt/β-catenin, Hedgehog, TGF-β/SMAD, JAK/STAT, NF-κB; many growth factors, including interleukins 6 and 8; and proteins such as E/N-cadherins and vimentin [22,107] (Figure 1). Preclinical findings have provided clear evidence that resistance to trastuzumab and lapatinib can occur spontaneously following EMT as HER2+ BC cells shift from a luminal to a basal/mesenchymal phenotype with high enrichment of CD44^high^/CD24^low^ BCSCs and inherent EMT traits [108]; specifically, preclinical evidence has highlighted a relevant increase in Slug, Twist1 and ZEB1 expression levels in trastuzumab-refractory basal/HER2+ cells versus trastuzumab-susceptible luminal/HER2+ cell models [109].

## 5. Summary of Actionable HER2+ BCSC Vulnerabilities

The majority of currently available anti-CSC treatment strategies aim to target stemness-associated traits; however, a major limitation in their design and therapeutic efficacy is that the majority of these stemness-associated traits are shared between CSCs and normal stem cells [110]. In addition, in contrast to previous antitumor therapeutic approaches, new treatments need to consider strategic combinations that are able to kill CSCs and bulk tumor cells, as well as to prevent the transition of highly proliferating bulk cells into quiescent CSCs [35]. Emerging therapies targeting a plethora of CSC vulnerabilities under evaluation as single-agents or in combination with conventional chemotherapy in numerous solid and hematological malignancies have been recently summarized [110]. Specifically, concerning HER2+ BC, and as already outlined before, the currently selected emerging therapies designed to overcome trastuzumab resistance aim to target critical downstream signaling molecules whose dysregulation is significantly implicated in sustaining oncogenic HER2 signaling and the maintenance/survival of BCSCs. In particular, as listed in Table 1, many preclinical studies targeting either ALDH+ or CD44^high^/CD24^low^ BCSCs and/or both rely on the targeting of: HER2/AKT signaling [48,49], PI3K/AKT signaling [24,111,112,113], TGF-beta signaling [99,100,114], JAK/STAT signaling [115], and Hippo signaling [116]. These strategies are currently under evaluation in HER2+ trastuzumab-resistant models, either in combination with the biodrug or without combination. Other actionable CSC-related vulnerabilities whose targeting by monotherapy or trastuzumab combination therapy is implicated in the reduction/eradication of CD44^high^/CD24^low^/ALDH+ HER2+ BCSCs include the IL6 autocrine loop [113,117], IL8/CXCR1/2 signaling axis [118], MUC1 [119], N-glycosylated β1-integrin [108], CDK12 [96], MEOX1 [120] and the d16HER2 splice variant [78] (Table 1).

Further investigations evaluating the interaction between CSCs and the immune system and clinical applications of targeting CSCs using immunotherapeutic approaches (in particular CAR T cells) can provide promising future therapeutic perspectives in the context of HER2+ BCs [121].

**Table 1:** Targeting of BCSCs in HER2+ BC models refractory to trastuzumab treatment.

BCSC Targets	Drugs	Reference
HER2/AKT	Disulfiram	[48]
	Flubendazole	[49]
PI3K/AKT	XL147	[111]
	NVP-BKM120	[112]
	LY-2940002	[24]
	Perifosine	[113]
TGF-β signaling	SIS3	[99]
	A83-01	[100]
	Metformin	[114]
Hippo signaling	Verteporfin	[116]
IL-6 autocrine signaling	anti-IL-6 receptor Ab	[113]
	MEDI5117	[117]
IL8/CXCR1/2 signaling	SCH563705	[118]
MUC1	AZD1775/WEE1 Inhibitor	[119]
N-glycosilated β1-integrin	AllB2 antibody	[108]
CDK12	Dinaciclib	[96]
MEOX1	Sulforaphane	[120]
d16HER2 splice variant	Isothiocyanate (PEITC)	[78]
HSP90	NCT-547	[50]
ADAM10	INCB8765	[103]

## 6. Conclusions

Seminal studies have highlighted the key role played by altered HER2 signaling in the maintenance/enrichment of BCSCs, and HER2+ BCSC reduction has been shown to restore trastuzumab susceptibility in relevant in vitro and in vivo models. Of note, although preclinical stem-cell-based therapies have demonstrated considerable potential as novel anticancer approaches, most putative anti-CSC therapies to date have demonstrated limited efficacy in the clinical setting. In this context, it is important to note that different signaling pathways crosstalk with HER2 and can crosstalk with each other, thus complicating the pathobiological scenario of HER2+ BC. To develop a cure aimed at eradicating the “root” of cancer, we believe that the most important challenges to be addressed include the identification and targeting of the central node orchestrating the multiple and very complex signaling networks responsible for the maintenance/proliferation of BCSCs and their crosstalk with the surrounding microenvironment, and an understanding of the mechanisms underlying CSC plasticity, since CSCs can transition between CSC and non-CSC state and vice versa. Additionally, the clinical applicability of the CSC concept for predicting patient response remains unknown, and new clinical trials will have to be developed to assess the therapeutic efficacy and safety of these therapies. Thus, to improve the evaluation of the efficacy of anti-CSC agents in clinical trials, there is a pressing need to optimize the specific response criteria that are used to determine the response to anti-CSC agents. In fact, tumor regression, a currently used clinical endpoint, is inadequate when CSCs constitute only the minority of the cells within a solid tumor. An increased understanding of all these aspects might help in the development of more effective therapeutic strategies and in the achievement of durable responses.
